# The influence of emotional stimuli on response inhibition: a systematic review in non-clinical adults

**DOI:** 10.3389/fpsyg.2025.1577486

**Published:** 2025-05-06

**Authors:** Irene Rincón-Pérez, Alberto J. Sánchez-Carmona, David Levy, Sara López-Martín, José A. Hinojosa, Jacobo Albert

**Affiliations:** ^1^Facultad de Psicología, Universidad Autónoma de Madrid (UAM), Madrid, Spain; ^2^Facultad de Psicología, Universidad Complutense de Madrid (UCM), Madrid, Spain; ^3^Neuromottiva, Madrid, Spain; ^4^Instituto Pluridisciplinar, Universidad Complutense de Madrid (UCM), Madrid, Spain; ^5^Centro de Investigación Nebrija en Cognición (CINC), Universidad Nebrija, Madrid, Spain

**Keywords:** response inhibition, emotion, Go/No-Go task, stop-signal task, valence, arousal, task relevance, systematic review

## Abstract

This PRISMA-compliant systematic review aimed to clarify the influence of emotional stimuli on the behavioral correlates of response inhibition, given the mixed and inconclusive findings in the existing literature. We searched Scopus, PsycINFO and PubMed databases up to March 2024 for studies published in peer-reviewed journals, conducted in adult non-clinical populations. Eligible studies used tasks where response inhibition plays a central role (primarily the Go/No-Go task [GNG] and stop-signal task [SST]) and included emotional stimuli presented concurrently with the task. Additionally, studies had to report, control for, analyze, or at least discuss both valence (positive-negative) and arousal (calming-arousing), two emotional dimensions that have been widely used to define emotions. Ninety-three studies, encompassing over 3,400 participants, were finally included, and assessed using the Appraisal tool for Cross-Sectional Studies (AXIS). Most studies report emotional modulation of response inhibition, with a larger proportion linking this influence to valence rather than arousal. However, inconsistent findings and methodological limitations prevent firm conclusions, with some suggesting the involvement of both or neither dimension, while others lack the appropriate design. Among studies reporting effects of emotional valence, most indicate that higher valence stimuli (more positive) impaired response inhibition. The effects of arousal remain unclear, with some studies linking high arousal to poorer inhibition, while others suggest the opposite. Interestingly, discrete emotions also modulate response inhibition independently of valence and arousal, suggesting that discrete emotion theories may complement the two-dimensional circumplex model in response inhibition research. While few differences exist, more studies report effects when emotional stimuli are task-relevant rather than task-irrelevant. Among other factors, using an SST instead of a GNG task seems to enhance emotional modulation of response inhibition. Overall, the influence of emotional stimuli on the behavioral correlates of response inhibition is likely shaped by a complex interplay of multiple factors, suggesting that future research should explore how these factors interact and combine. Moreover, further research is needed to explore how emotion interacts with other forms of inhibitory control beyond global reactive inhibition, including proactive and selective mechanisms.

## 1 Introduction

Inhibitory control is a fundamental executive function crucial for adaptive behavior and cognition (Diamond, 2013). It has traditionally been divided into *cognitive inhibition*, which involves suppressing mental processes such as memories and thoughts, and *behavioral inhibition*, which refers to overriding or stopping observable actions (Bari and Robbins, 2013). Response inhibition (the ability to suppress actions that are inappropriate, unsafe or no longer required) is undoubtedly the most studied type of behavioral inhibition. Effective response inhibition is fundamental for maintaining goal-directed and flexible behavior in dynamic, ever-changing environments. Indeed, difficulties in response inhibition negatively impact functioning and quality of life in the general population (Diamond, 2013) and even more markedly in individuals with clinical disorders characterized by impulsive behaviors and deficits in inhibitory control, such as attention deficit hyperactivity disorder (ADHD), borderline personality disorder (BPD), or obsessive-compulsive disorder (OCD) (Albert et al., 2019; Slaats-Willemse et al., 2003; Mar et al., 2022; Menzies et al., 2007).

The go/no-go task (GNG) and the stop-signal task (SST) are the most commonly used paradigms to examine response inhibition in experimental settings (Verbruggen and Logan, 2008; Verbruggen et al., 2019), although tasks with lesser inhibitory demands exist (Wöstmann et al., 2013). Both tasks require controlling a prepotent motor response tendency but differ in the timing and nature of the response suppression: whereas the GNG involves withholding a response before it is initiated, the SST requires overriding an already initiated response (Raud et al., 2020; Schachar et al., 2007). Moreover, each task provides a distinct behavioral index of response inhibition: commission errors in the GNG task and stop-signal reaction time (SSRT) in the SST. Commission errors (i.e., inappropriate responding to No-Go stimuli) are usually interpreted as a failure in prepotent response inhibition. A higher number of commission errors would therefore reflect poorer inhibitory control. By contrast, SSRT is an estimation of the response-inhibition latency (i.e., the time needed to cancel the initiated response, which cannot be directly observable). Thus, longer SSRTs are typically associated with less efficient response inhibition and higher impulsivity in large samples from the general population (Crosbie et al., 2013; Moses et al., 2022).

Response inhibition elicited by standard versions of the GNG and SST is thought to be mediated by a monosynaptic, hyperdirect pathway between the inferior frontal gyrus (IFG) and/or pre-supplementary motor area (pre-SMA) and the subthalamic nucleus (Chen et al., 2020; Narayanan et al., 2020). Activation of this fronto-subthalamic network has been associated with a rapid, stimulus-driven, and global suppression mechanism (Aron, 2011). Moreover, oscillatory activity in the ß frequency band (~13–29 Hz) and the onset of the event-related potential (ERP) component known as No-Go/Stop P3 have been proposed as robust neural signatures of response inhibition at the electrophysiological level (Albert et al., 2013; Hervault et al., 2025; Sánchez-Carmona et al., 2016, 2019; Wagner et al., 2018; Wessel and Aron, 2015).

Most research on response inhibition has focused on elucidating the neural and behavioral mechanisms involved in suppressing motor responses to neutral, non-salient stimuli. These investigations have provided extensive and crucial knowledge, enabling the development of the most influential models of response inhibition (Aron, 2011; Schall et al., 2017). In many real-world scenarios, however, individuals must inhibit their impulses and responses to emotionally charged stimuli, whether negative (e.g., seeing an angry expression on someone close to you or hearing a loud noise, like a car horn) or positive (seeing an attractive person or hearing great news). Understanding how emotion interacts with response inhibition is therefore crucial for expanding current models of inhibitory control in humans. However, studies examining the emotional modulation of response inhibition report mixed and inconsistent findings, with some even suggesting that emotion does not influence inhibitory control depending on the circumstances (e.g., Harlé et al., 2013; Pessoa, 2009; Schel and Crone, 2013; Shafritz et al., 2006; Williams et al., 2020).

The effect of emotion on response inhibition has primarily been studied using the two-dimensional circumplex model (Russell, 1980, 2003). This model defines emotions along two continuous dimensions: valence, which ranges from pleasant (positive) to unpleasant (negative), and arousal, which reflects the level of activation from calming to exciting. Some studies using this theoretical framework suggest that the modulatory effect of emotions on response inhibition is mediated by the valence of stimuli, while others propose that emotional effects are driven by arousal (Verbruggen and De Houwer, 2007). The direction of the effects related to emotional valence is also unclear, with evidence suggesting impaired inhibitory control (i.e., more commission errors and/or prolonged SSRT) in response to negative stimuli compared to positive ones, and vice versa (e.g., see Fournier et al., 2021; Gupta and Singh, 2021; Xia et al., 2018; Zhang J. et al., 2023). With respect to the arousal dimension, evidence suggests both impairment and facilitation of response inhibition for high-intensity stimuli, regardless of their valence (Pessoa et al., 2012; Verbruggen and De Houwer, 2007). Therefore, a systematic review of the impact of emotional stimuli on response inhibition, such as the one presented here, can help clarify the interplay between emotion and response inhibition by considering studies that adequately control for valence and arousal.

It should be noted that some investigations using emotional response inhibition tasks have been conducted within a conceptual framework different from the dimensional model of emotions. Specifically, these studies -primarily using emotional facial expression, though not exclusively- are grounded in discrete-emotion theories (Ekman, 1992; Panksepp and Watt, 2011). These models propose that emotional effects stem from a limited number of innate and universal emotions, each linked to distinct and independent behavioral, psychological, and physiological correlates. From this perspective, the emotional modulation of response inhibition may differ between stimuli typically classified as negative (e.g., fear, anger, disgust or sadness) or positive (love, pride, gratitude or happiness), as suggested by Storbeck et al. (2024). However, these differences in the modulatory effects of discrete emotions on response inhibition could also be explained through the dimensional model if valence and arousal levels are not properly controlled. For instance, if the influence of discrete negative emotions, such as fear and disgust, on response inhibition is examined using stimuli that differ in valence or arousal, the results may reflect these dimension-related variations rather than the specific effects of each emotion.

Another key factor that seems to modulate the influence of emotion on cognitive functions is whether the emotional content of the stimuli is processed in a relatively unintentional implicit fashion (task-irrelevant emotional stimuli) or in a controlled explicit manner (task-relevant emotional stimuli). In other words, this distinction depends on whether the emotional content of the stimuli serves as an explicit criterion for task completion (Battaglia et al., 2021; Yuan et al., 2019). Emotional stimuli are known to automatically capture attention, even in non-emotional tasks (Mulckhuyse, 2018; Pool et al., 2016; Shafer et al., 2012). As a result, competition for cognitive resources may interfere with task goals (Pessoa, 2009). Conversely, explicitly directing attention to emotion can be beneficial when fast, goal-related affective processing is required, as emotional stimuli are detected and processed faster than non-emotional ones (Brosch et al., 2010), and can influence the speed of movement initiation and response execution (Beatty et al., 2016). Thus, both mechanisms may play a role in modulating the interaction between emotion and response inhibition. Specifically, in response inhibition paradigms, the use of emotionally relevant stimuli for the task implies that attention is directed specifically toward the emotional properties of the stimuli (e.g., asking participants to respond to happy faces and stop their responses to fearful ones). By contrast when emotionally irrelevant stimuli are used in response inhibition tasks, attention is directed toward the non-emotional features of the stimuli (e.g., asking subjects to inhibit their response to a specific physical feature of emotional stimuli, such as the color of the image border or the type of font in words). Therefore, emotion may modulate response inhibition either implicitly or explicitly. Notably, several studies examining both task-relevant and task-irrelevant emotional aspects of stimuli have found effects primarily when emotion is relevant to the task (e.g., Calbi et al., 2022; Mancini et al., 2022). However, some studies have also found no effect when emotional stimuli are task-relevant (Schmaußer and Laborde, 2023; Zhang et al., 2016), which further contributes to the mixed findings. In any case, the task relevance of the emotional content of the stimuli appears to be an important factor in the emotional modulation of response inhibition, as previously suggested in a review of studies using emotional versions of the SST task (Battaglia et al., 2021), along with valence and arousal. To further expand our understanding of emotional response inhibition, a broader review incorporating other inhibitory tasks and additional influencing factors beyond task relevance is essential.

Given the mixed findings in the literature on the emotional modulation of response inhibition and the fact that several key questions remain elusive, we conducted a PRISMA-compliant systematic review to examine the influence of emotion on behavioral measures of response inhibition in non-clinical adult samples. Specifically, the objectives were as follows: (1) to investigate whether the emotional content of stimuli modulates the main behavioral correlates of response inhibition; (2) to examine whether the emotional modulation of response inhibition is related to valence (pleasantness-unpleasantness), arousal (calming-arousing), or both; (3) to explore the direction of the effects within each emotional dimension: whether impairment in response inhibition is observed in response to positive (pleasant) vs. negative (unpleasant) stimuli (when emotional modulation is mainly associated with valence) or to high- versus low-intensity stimuli (when emotional modulation is primarily related to arousal); (4) to examine whether the task relevance of the emotional content of stimuli influences response inhibition; (5) to investigate other factors that may influence the emotional modulation of response inhibition, such as the type of inhibition task used (GNG or SST) or the type of emotional stimulus employed (pictures, faces, words or sounds; Brosch et al., 2010; Yuan et al., 2019).

## 2 Methods

### 2.1 Data sources and search strategy

This systematic review was conducted following PRISMA guidelines (Page et al., 2021). Searches were performed in the Scopus, PsycINFO and PubMed databases, chosen for their wide coverage and/or their complementary scope (Bramer et al., 2017). In each database, we first created a search string combining the terms “emotion,” “emotional,” “affective stimuli,” and “emotional stimuli” with “response inhibition,” “inhibitory control,” “stopping,” “response suppression,” or “action cancellation.” Additionally, we constructed a second search string by incorporating terms related to response inhibition tasks to ensure comprehensive coverage of relevant results. Thus, the final search term combination was as follows: (“emotion” OR “emotional” OR “affective stimuli” OR “emotional stimuli”) AND (“response inhibition” OR “inhibitory control” OR “stopping” OR “response suppression” OR “action cancellation” OR go no go task OR stop signal task OR CPT OR SART). Where available, filters were applied to include only manuscripts in English and Spanish, focusing exclusively on adult populations and peer-reviewed publications. These searches were conducted up to March 2024 (included).

### 2.2 Systematic review protocol

After all records were downloaded and duplicates were removed (using EndNote software), we visually inspected the remaining records for any duplicates the automated process might have missed. Then, we screened titles and abstracts and applied the following exclusion criteria: (1) the record was not a peer-reviewed scientific full article published in an indexed journal (papers without results such as pre-registered trials were excluded, theses were excluded, letters to the editor were excluded, papers in journals with unclear indexing were excluded); (2) the record did not pertain to human adults: given that emotion recognition declines with age (Ruffman et al., 2008) and that children and adolescents experience dramatic changes in emotion dynamics and experience before reaching adulthood (Bailen et al., 2019; Reitsema et al., 2022), we decided to limit the study population to adults in order to mitigate these discrepancies; (3) the record elicited emotions through approaches other than presenting emotional stimuli concurrently with the response inhibition task; (4) the record did not pertain to response inhibition; (5) the record did not include a general population group. In order to reduce potential sources of heterogeneity that could bias the characterization of emotional modulation of response inhibition in the general population, we excluded studies with samples characterized by current or recent affective clinical difficulties, such as the following: hemianopia populations, postpartum mothers when there were high depression symptoms, war veterans when there was high post-traumatic stress disorder presence, partially recovered depression populations, and relapsed alcohol abstainers. We also excluded studies where, although the samples were drawn from the general population, closer inspection revealed a high percentage of participants with a clinical disorder such as substance abuse disorder, anxiety or depression that were not controlled for in the analyses. For example, studies involving soldiers in training due to high anxiety/stress or individuals traumatized by an earthquake; (6) the record was not written in English or Spanish.

After the initial screening, we retrieved the full-text of the remaining papers (or asked authors for the full-text if it was not available to us). There was one record we were not able to retrieve. During the full-text assessment phase of the process, we excluded records if: (1) no emotional stimuli were presented concurrently with the response inhibition task (studies where stimuli were presented prior to a non-emotional task, such as mood inductions, were excluded. Although mood inductions may be considered task-irrelevant, we considered it was inappropriate to compare them with task-irrelevant stimuli presented during response inhibition tasks, as the attention resources allocated to emotional stimulation would differ in each case. All other emotional inductions without explicit mention of containing emotional stimuli, such as thinking or writing about negative life events, and fear conditioning procedures were also excluded. However, if the study included a control condition for the emotional induction as well as an emotional inhibition task, it was considered for inclusion. The diversity in emotional inductions was another reason for exclusion, as it would introduce excessive heterogeneity. Additionally, for consistency, we only included studies where the emotional stimuli were concurrent with the task, therefore studies in which the presentation of the emotional stimuli occurred between the practice and the test blocks were also excluded); (2) the study did not use a task where response inhibition is predominant (e.g., Stroop, Day-Night, Flanker, Oddball, Antisaccade, Dot-Probe tasks were excluded. Additionally, studies that did not involve the inhibition of a manual motor response (e.g., suppression of smiling or eye movements) were excluded, given the limited number of studies for each type of these motor responses. We also considered that the influence of emotion on these responses might differ from what has been observed with manual motor responses, which are undoubtedly the most studied in response inhibition research. If motor responses to emotional stimuli (such as facial movements) were made with the mouth corners, we considered that facial mimicry might obscure response inhibition results and thus excluded such studies. While we included studies using tasks where response inhibition was predominant, we excluded one study (Windmann and Chmielewski, 2008) due to the high memory load of the task used, which we considered a potential confound); (3) there was no information reporting the effects of emotional stimuli on behavioral correlates of response inhibition (such as when the focus was on differences between healthy and clinical groups, or between treatment and no treatment groups); (4) there was an intervention without a control condition; (5) neither valence nor arousal were reported, controlled for, included in the analysis, or at least discussed by the authors (note that if a study included multiple tasks but valence/arousal data were not provided for the stimuli used in all tasks, we only considered the tasks that included such data); (6) the study used response inhibition tasks but did not report any behavioral or neural correlate of response inhibition (e.g., studies that analyzed only Go response times, or studies that examined only ERP components unrelated to response inhibition).

All studies included in this review examine behavioral correlates of response inhibition tasks, with some additionally exploring neural correlates using electroencephalography (EEG) and/or hemodynamic (fMRI) measures. Studies that met our inclusion criteria based on their behavioral data were included. However, neural findings were excluded from our summary of results if the studies using these techniques did not report neural data segregated by emotion or an appropriate contrast comparing emotions and/or if they reported ERP components unrelated to response inhibition (such as early face processing or late evaluation components), or if the results were not reported for a control group. In addition, behavioral studies that did not explicitly report response inhibition behavioral correlates such as commission errors and/or SSRT were also excluded (e.g., if a study focused the analysis on signal detection theory or if it only reported omission errors and Go-trial response times), unless they reported additional EEG/fMRI results related to response inhibition.

The first author screened the records and retrieved papers, discussing any uncertainties with the last author before reaching a final resolution by consensus among all authors to minimize the risk of bias.

### 2.3 Data extraction

Data were extracted and coded by the first author, then reviewed by the last author, and finally by the remaining authors. The extracted data included the following: (1) General study information: the authors and the publication year of each study; (2) Details on the emotional stimuli used in each study: the type of emotional cues (words, pictures, faces, body postures or sounds), whether they were relevant or irrelevant to performing the response inhibition task, the different categories of stimuli as labeled by the authors, and information on valence and arousal of each category; (3) Methodological information: this included demographic data on the participants, whether the study was focused solely on behavioral methods or also incorporated electrophysiological or haemodynamic analytic approaches, and the type of response inhibition task used in the study; (4) Study results: this involved distinguishing between behavioral and brain activity results, as well as identifying any relevant variables noted by the authors that might explain the results.

Given the central role of valence and arousal in this review, we introduced an additional variable to describe whether observed effects were driven by valence, arousal, both, neither, or if they were unclear. When the study design made it difficult or impossible to distinguish these dimensional effects, this was noted. Additionally, we introduced a variable to describe the direction of the effects, identifying which level of valence or arousal was associated with poorer response inhibition at the behavioral level. This was applied to investigations where a clear driver of the behavioral effect was identified, with the effects categorized as high or low valence/arousal, or deemed unclear.

We also included the type of inhibitory task (GNG or SST) as a variable because these two paradigms are thought to rely on different mechanisms and capture distinct inhibitory processes (action restraint and action cancellation, respectively; Aziz-Safaie et al., 2024; Raud et al., 2020; Schachar et al., 2007). Therefore, we considered it important to explore whether emotional stimuli might have differential effects depending on the task used.

It is worth noting that some studies analyzed and presented their results in ways that did not fully align with the scope of this review, with only certain parts deemed relevant. For instance, some studies focused on patient-control differences or outcomes of an intervention. In such cases, only the data relevant to our objectives and reported in full were included (see Supplementary Table 1).

### 2.4 Quality assessment

The appraisal tool for cross-sectional studies (AXIS) was used to assess the quality of each study included in this systematic review (Downes et al., 2016). Briefly, AXIS examines the reliability, risk of bias and quality of studies through 20 items covering the following aspects: study design, sample selection, variable measurement, bias control, statistical analysis, and the relevance of results. Studies deemed to be of insufficient quality according to AXIS were excluded (defined as less than 14 out of 20 items in the tool answered with a “yes”, except for item 19 which should be answered with a “no”).

### 2.5 Data analysis

Data were descriptively analyzed using MS Excel and JASP (0.19.2).

## 3 Results

### 3.1 Flow diagram, study selection and characteristics

**Figure 1:**
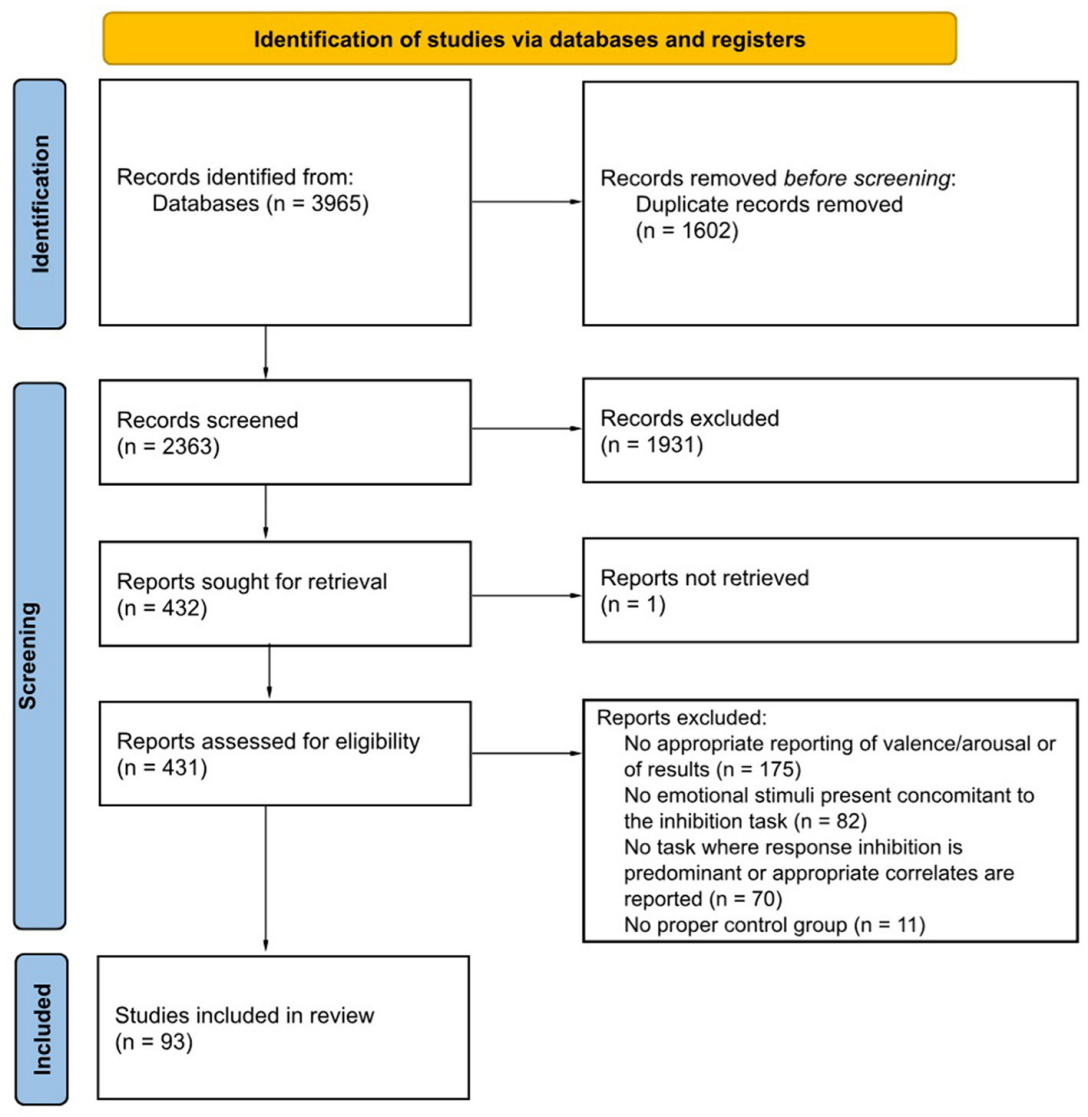
PRISMA 2020 flow diagram representing the review process. Adapted from Page et al. (2021) and Haddaway et al. (2022).

From an initial total of 3,965 records, 2,363 remained after duplicate removal and were screened, of which 1,931 were then excluded (see Section 2.2). Afterwards, 432 records were identified for full-text assessment, with one record that could not be retrieved. From the resulting 431 records, 93 records met all the inclusion/exclusion criteria, as shown in Figure 1. A summary of all selected papers is presented in Table 1. However, readers are encouraged to also consult Supplementary Table 1 for a more detailed overview, including a comprehensive breakdown of study characteristics.

**Table 1:** Summarized overview of included studies.

**Were stimuli task-relevant?**	**Study found behavioral results?**				
	**Y**				**N**
	**Driver of behavioral effects?**				
	**Valence**	**Arousal**	**Both/Unclear**	**Impossible to distinguish**	
Y	Allen and Hooley, 2019; Amin et al., 2006	Yang et al., 2014	Chiu et al., 2008; Greif and Waring, 2018; van Holst et al., 2012a,b; Vercammen et al., 2012	Ding et al., 2020; Jia et al., 2023; Song et al., 2016; You et al., 2020; Zhang X. et al., 2023	Berlin et al., 2015; García-Blanco et al., 2013; Liu et al., 2018; Ma et al., 2013; Schmaußer and Laborde, 2023; Sun et al., 2020; Vercammen et al., 2013; Zhang et al., 2016
Both	Calbi et al., 2022; Mancini et al., 2022; Yu et al., 2014^*^	-	-	Gole et al., 2012	-
N	Albert et al., 2012; Andreu et al., 2019; Benvenuti et al., 2015; Buodo et al., 2017; Fournier et al., 2021; Gupta and Singh, 2021; Liu et al., 2021; Pandey and Gupta, 2022; Zhang J. et al., 2023; Zhang et al., 2020; Xia et al., 2018	Battaglia et al., 2022b; Pessoa et al., 2012; Verbruggen and De Houwer, 2007	De Houwer and Tibboel, 2010; Demers et al., 2022; Gupta and Singh, 2023; Jones and Field, 2015; Lodha and Gupta, 2024; Mennella et al., 2017; Sitges et al., 2018; Su et al., 2022; van Zutphen et al., 2020; Wiemer et al., 2023; Wolz et al., 2021; Zheng et al., 2020	Battaglia et al., 2022a; Kalanthroff et al., 2013; Kampa et al., 2023; Krypotos et al., 2011; Littman and Takács, 2017; Senderecka, 2016, 2018; Verona et al., 2012; Wilson et al., 2016; Xu et al., 2016b; Yu et al., 2012, 2015; Zhuang et al., 2021	Agudelo-Orjuela et al., 2021; Albert et al., 2010; Asci et al., 2019; Atkinson-Clement et al., 2020; Benvenuti et al., 2017; Brown et al., 2012, 2015; Camfield et al., 2018; Chester et al., 2016; Cohen-Gilbert and Thomas, 2013; Contreras et al., 2013; De Sanctis et al., 2013; Fink-Lamotte et al., 2021; Kakuszi et al., 2020; Mallorquí-Bagué et al., 2020; Moretta and Buodo, 2021; Morie et al., 2014; Ramos et al., 2024; Ramos-Loyo et al., 2016, 2021; Senderecka et al., 2018; Stockdale et al., 2020; Todd et al., 2012; Xu et al., 2016a, 2015; Yu et al., 2009; Zhang et al., 2012; Zhang and Lu, 2012; Zhao et al., 2019

Please refer to Supplementary Table 1 for more information. ^*^It should be noted that the study by Yu et al. (2014) did not report behavioral effects, but did find brain activity effects related to valence. Y = Yes, N = No.

All selected papers were written in English and published between 2006 and 2024. Most were published since 2012, with the highest numbers recorded in 2012 (*N* = 12) and 2020 (*N* = 10). Notably, 41.9% were published from 2019 onwards, reflecting a recent increase in interest.

All selected articles were published in indexed, peer-reviewed journals within the fields of psychology, neuroscience, and/or medicine, except for those published in *PLoS ONE* and *Scientific Reports*, which are considered multidisciplinary. The journal with the highest number of articles was *PLoS ONE* (*N* = 7), followed by *NeuroImage* (*N* = 6). Most studies were conducted in Western countries and China.

### 3.2 Synthesized findings

#### 3.2.1 Behavioral results

The main characteristics and key findings of each study included in this systematic review are presented in Supplementary Table 1. After the screening process, all studies included in the final review used either the Go/No-Go (GNG) task (63 studies) or the Stop-Signal Task (SST; 28 studies), except for one study that combined both tasks and another that used a Sustained Attention to Response Task (SART), which was notably similar to the GNG. Of the 93 studies included in this review, 55 (59.1%) reported an effect of emotional cues on the behavioral correlates of response inhibition (i.e., commission errors and/or SSRT).

Out of the 55 studies showing emotional effects on behavioral correlates of response inhibition, 15 studies (27.3%) suggested that these effects were influenced by valence, 4 studies (7.3%) by arousal, 17 studies (30.9%) showed unclear results (where both or neither emotional dimensions could have been involved), and in 19 studies (34.5%) it was not possible to discern due to the study designs not accounting for such a purpose. Among the 15 studies in which effects related to emotional valence were observed, higher valence (more positive) was associated with poorer response inhibition in ten of them (66.7%). The results of the 4 studies that observed effects related to emotional arousal are mixed, with higher arousal being associated with both impaired (in two studies) and enhanced (in the other two studies) response inhibition.

Most studies have examined the influence of emotion on response inhibition using emotional stimuli that were irrelevant to the ongoing task (68/93 studies). Thirty-nine of these studies (57.4%) showed some effect of emotion on response inhibition at the behavioral level. Of the 21 studies that explored the emotional modulation of response inhibition using stimuli relevant to the ongoing task, thirteen (61.9%) showed emotional effects on the behavioral correlates of response inhibition. Interestingly, the three studies that tested behaviorally both emotionally task-relevant and task-irrelevant stimuli embedded in the same response inhibition paradigms (Calbi et al., 2022; Gole et al., 2012; Mancini et al., 2022) observed effects when the emotional aspects of the stimuli were task-relevant (there were more errors in the emotional tasks or blocks). This somewhat contradicts the overall trend of the review, where effects are only slightly more likely to be observed when the emotional aspects of the stimuli are task-relevant (61.9 vs. 57.4%).

Regarding the type of stimuli used in the studies, the most frequently employed were pictures (53 studies), either as backgrounds (17 studies) or as cues to which participants had to respond directly (36 studies) either attending to the emotion or to some other characteristic of the stimuli. Human faces were used in 23 studies, while words appeared in 14 studies. Sounds and body postures were the least frequent (2 and 3 studies respectively) and therefore insufficient to draw strong conclusions. No marked differences were observed in the percentage of studies finding emotional effects on behavioral correlates of response inhibition based on the type of stimulus used. Overall, finding an effect of the emotional stimuli on response inhibition at the behavioral level was more likely than not, and occurred at similar rates across all stimulus types (ranging from 65.2 to 71.4%). There was one notable exception: when pictures were used as a background, it was much more likely to not find an emotional effect (76.5% of studies that used pictures as a background did not find one).

With respect to the type of task used, among the 63 studies employing a GNG task, 30 (47.6%) found an effect of emotional stimuli on response inhibition at the behavioral level, 32 (50.8%) did not, and one reported effects only at the neural level. The study that used a SART (which was similar to a GNG) also reported effects. In contrast, among the 28 studies using an SST, the majority (82.1%) observed an effect of the emotional stimuli on response inhibition at the behavioral level. The study that used a mixed GNG-SST design also reported such an effect. Notably, a number of studies employing GNG tasks (13 out of 63) were designed with a 50% frequency of No-Go stimuli, reducing the prepotency of Go responses (since Go and No-Go trials occurred equally often) and thereby lowering the task's inhibitory demands. This design choice may explain why a substantial percentage of these experiments (9 out of those 13; 69.23%) found no effect of emotional stimuli on response inhibition at the behavioral level. Among the remaining 50 GNG studies, which included lower frequencies of No-Go stimuli (resulting in higher prepotency and greater inhibitory demands), findings were evenly split: 46% reported no effect, 52% found an effect, and 2% did not report behavioral correlates. Nonetheless, even when considering only GNG studies with a low percentage of No-Go stimuli, the proportion of investigations reporting emotional effects on response inhibition at the behavioral level remains notably lower compared to SST experiments.

When attempting to examine the behavioral effects of emotion on response inhibition by combining these variables, the resulting subgroups were too small to draw definitive conclusions. Nonetheless, no clear pattern emerged when examining interactions between factors.

Some of the studies reviewed here propose additional factors that may influence the affective modulation of response inhibition. The most frequently mentioned factors were related to underlying traits of the sample, followed by differences in sex or age (see Supplementary Table 1 for details). Less frequently, specific properties of the stimuli or of the way they are presented in the task (e.g., masking, timing or perceptual load) were also noted.

#### 3.2.2 Neural results

Although it was not the main aim of this systematic review, we conducted an exploratory *post-hoc* analysis of the influence of emotional stimuli on response inhibition at the neural level in behavioral studies that also included brain activity measures, such as EEG and fMRI.

Forty-eight studies included in this review examined the emotional effects on response inhibition not only at the behavioral level, but also at the neural level. Most of these studies (39 studies; 81.2%) found effects of emotional stimuli on neural activity associated with response inhibition, using either electrophysiological or hemodynamic measures. Notably, emotional effects on response inhibition at the neural level were often observed even in the absence of behavioral effects. Neuroimaging results reveal activation in several regions associated with both response inhibition and emotional processing, as expected (see Supplementary Table 1 for details). Additionally, the involvement of some cortical regions previously linked to the interaction between emotion and cognitive control processes, such as the orbitofrontal cortex and anterior cingulate cortex, was observed. Electrophysiological findings indicate amplitude differences in No-Go/Stop N2 and No-Go/Stop P3, which are typically obtained in response inhibition tasks.

The effects of the emotional content of the stimuli on neural correlates of response inhibition were associated with both valence and arousal dimensions. However, in the same way as in the behavioral findings, a greater number of studies found that emotional modulation is associated with valence (14/39 studies, 35.9%) rather than arousal (6/39 studies, 15,4%). In the remaining studies, either the experimental designs or the observed results made it difficult to discern which emotional dimension (valence or arousal) accounted for the observed effects. Furthermore, the direction of results is unclear, much like for the behavioral results. There was no consensus on the direction of effects, given that in many cases effects were opposite for No-Go/Stop N2 and No-Go/Stop P3 (or one showed an effect and the other did not). Separation by No-Go/Stop N2 or P3 did not yield clear results either, as sometimes a given valence or arousal category was associated with higher amplitudes, and sometimes the opposite valence/arousal gave similar results (see Supplementary Table 1 for details). Neuroimaging results revealed a plethora of regions activated differently according to the emotional stimuli used (note that activation in different areas does not necessarily indicate better or worse inhibitory ability. Therefore, where applicable, this is marked as “N/A” in the corresponding column of our Supplementary Table 1).

The task relevance of emotional stimuli does not appear to be a decisive factor in generating emotional effects on inhibition-related neural activation, as similar proportions of studies reported effects whether the emotional content of the stimulus was relevant (10 out of 12; 83.3%) or irrelevant (28 out of 35; 80%) to the ongoing task. It is worth noting, however, that the only brain activation study to include both relevant and irrelevant emotional stimuli within the same response inhibition experiment found effects only when the emotional content was relevant to task completion (Yu et al., 2014).

The types of stimuli used did not seem to have a significant effect either. The majority of studies observe emotional modulation of response inhibition at the neural level regardless of the type of stimulus used (faces, images, or words; emotional effects found in the 72.7–90% of studies). Sounds were used rarely (only 2 studies), making it difficult to draw conclusions. Regarding the type of task, emotional modulation of neural activation associated with response inhibition is found in most studies using both the SST and GNG tasks. However, in contrast to behavioral findings, the number of studies reporting emotional effects on neural activity related to inhibition is slightly higher when using a GNG task (82.9%) compared to an SST (71.4%).

### 3.3 Assessment of risk of bias

All of the 93 Studies included in the final step of the review were deemed of sufficient quality to be included, no study was excluded for quality reasons (see Supplementary Table 1).

## 4 Discussion

This PRISMA-compliant systematic review was conducted to try to clarify the emotional modulation of response inhibition, given the mixed and inconclusive findings in the existing literature. By synthesizing evidence from studies examining behavioral correlates in non-clinical adult samples, we aimed to provide a clearer understanding of how emotional stimuli influence response inhibition.

For our first objective, the present findings suggest that emotion modulates response inhibition. Around 60% of the reviewed studies reported changes in behavioral indices of inhibitory control when emotional stimuli were included in the main response inhibition tasks, with a higher percentage observing modulatory effects in neural signatures. This result aligns with other lines of research that highlight the strong interdependence between emotion and cognitive processes, such as language, attention, memory and other cognitive control functions (Carretié, 2014; Hinojosa et al., 2020; Cromheeke and Mueller, 2014; Harlé et al., 2013). However, the precise nature of how emotional stimuli modulate response inhibition, which we sought to clarify through the subsequent objectives, has yet to be fully elucidated.

The relationship between emotion and response inhibition has primarily been explored using the two-dimensional circumplex model. In this context, it has been proposed that valence and arousal may be important factors in the emotional modulation of response inhibition (Battaglia et al., 2021; Harlé et al., 2013; Yuan et al., 2019). For our second objective, the evidence is mixed, with some studies suggesting that the emotional modulation of response inhibition is primarily related to valence, while others point to arousal. However, a greater proportion of experiments have found that this influence is associated with valence rather than arousal (27.3 and 7.3%, respectively). It should be noted, however, that the majority of studies included in this review do not allow for robust conclusions regarding which emotional dimension primarily modulates response inhibition. This is due to the lack of clear and consistent findings, with some studies suggesting the involvement of both or neither dimension, while others have methodological limitations that prevent a proper examination of this question. This review highlights the need for further studies in this field that carefully control for the valence and arousal levels of the stimuli used. For instance, in experiments using both negative and positive stimuli along with neutral ones, it is crucial to ensure that the arousal levels of the emotional stimuli are balanced and that they differ from those of neutral stimuli. Additionally, the valence of each type of emotional stimulus should be verified to ensure they are distinct from one another. A recommended approach is also to examine the relationship between the valence and arousal ratings of the stimuli—but obtained from the experimental sample—and the observed outcomes.

For our third objective, the findings from this review suggest that among studies reporting effects related to emotional valence, the majority indicate that higher valence (more positive) stimuli are associated with poorer response inhibition. Several studies have shown that positive contexts not only increase the number of commission errors but also lead to faster responses to Go stimuli (Albert et al., 2012; Hare et al., 2005; Zhuang et al., 2021; see also Mancini et al., 2022 and Mirabella et al., 2023). This pattern may suggest that positive valence may induce approach tendencies toward positive stimuli, making it more challenging to inhibit the prepotent response (Eder and Hommel, 2013). However, other studies have reported facilitated inhibition in response to positive valence (Pandey and Gupta, 2022). This suggests that the direction of effects within the valence dimension may vary depending on other factors outside the stimuli themselves, such as the way the stimuli are presented inside the task regarding masking, timing and perceptual load (Pandey and Gupta, 2022; Xu et al., 2015), likely due to competition for cognitive resources as we discuss below. On the other hand, the limited number of studies finding effects associated with arousal yields mixed results, with some indicating that higher arousal of stimuli impairs response inhibition, while others suggest that low arousal has this effect. These inconsistencies underscore the need for further research to clarify the direction of effects within each emotional dimension.

Moreover, it appears that certain emotions may be processed differently in the context of response inhibition, even when they are similar in valence and arousal to other emotions (Buodo et al., 2017; Mennella et al., 2017; Xu et al., 2015, 2016b), which supports the idea of a discrete emotions framework. This variation could be due to the biological significance of some stimuli, which have been shown to engage brain regions differently, likely due to their heightened biological and social relevance (Sakaki et al., 2012). Likewise, some particular stimuli seem to be processed differently in certain populations, which further supports an alternative approach to the dimensional model of emotions. As an example, it has been shown that social drinkers displayed more disinhibition during a modified SST in response to both alcohol and negatively valenced pictures, relative to both positive and neutral pictures (Jones and Field, 2015). In samples taken from the general non-diagnosed population, underlying characteristics of the sample such as worry-proneness (indicative of a generalized anxiety disorder) lead to more errors when worry-related words were present, compared to a low-worry group (Gole et al., 2012). Similar results occurred for angry words and athletes compared to non-athletes (Xia et al., 2018). Likewise, the regular practice of meditation also seemed to affect the processing of some categories of stimuli such as anger or happiness-related stimuli (Lodha and Gupta, 2024). Of note, these differences in disinhibition could not be completely accounted for by variations in arousal or valence ratings between stimuli sets.

Regarding our fourth objective, affective modulation of the behavioral correlates of response inhibition is observed both when the emotional content of the stimulus is relevant and when it is irrelevant to the task. However, a slightly higher percentage of studies report behavioral effects on response inhibition when the emotional content of the stimuli is relevant to the task than when it is not, which aligns with previous research (Battaglia et al., 2021). Moreover, the four studies that investigated emotionally task-relevant and task-irrelevant stimuli within the same experiment found effects only when the emotional aspects of the stimuli were task-relevant (Calbi et al., 2022; Gole et al., 2012; Mancini et al., 2022; Yu et al., 2014). This suggests a potential disparity between studies that directly contrast performance under both conditions (task-relevant vs. task-irrelevant) and those that examine only one. One possible explanation is that examining both conditions within the same experimental setting may reduce the influence of confounding variables, making the specific effects of task relevance more discernible. These findings may suggest that the facilitation of emotional stimuli processing also plays a role in response inhibition, as it does in other cognitive domains (Beatty et al., 2016; Brosch et al., 2010). This aligns with appraisal theories of emotion, which propose that affective stimuli produce different effects depending on how they are appraised. When emotional features align with task goals, they receive more attentional resources, enhancing the emotional response to the stimuli. Conversely, when they do not align, these features may be ignored in favor of other task-relevant information (Mancini et al., 2022; Moors and Fischer, 2019). In the same vein, a growing body of recent convergent evidence suggests that emotionally charged stimuli modulate various types of motor responses only when they are relevant to the ongoing task (e.g., forward gait initiation: Mirabella et al., 2023; saccadic responses: Mirabella et al., 2024; or reaching arm movements: Montalti and Mirabella, 2023). Accordingly, the task relevance of the emotional content of stimuli may influence response inhibition directly, as well as indirectly through its impact on response readiness.

We must consider that the relevance of emotion to the task is probably influenced by other factors, such as the cognitive load in which they are embedded and where and when the emotion is incorporated into the response inhibition task (Pessoa, 2009; Battaglia et al., 2021). In low-load tasks with task-irrelevant emotional stimuli, emotional effects may be more likely to emerge due to the absence of competition for cognitive resources. In contrast, high-load tasks with the same task-irrelevant stimuli may suppress these effects. Most of the studies reviewed here do not experimentally manipulate the cognitive load of the inhibition task, making it challenging to draw conclusions about its role in the emotional modulation of response inhibition, both independently and in interaction with task relevance. Therefore, further research is needed to explore the interplay between task-relevance of emotion and cognitive load in emotional response inhibition. Regarding the second factor, the effects of task relevance may vary depending on whether emotion is embedded into the go stimulus, the no-go/stop stimulus, both, or even before the presentation of the go stimulus (see Battaglia et al., 2021, for a review on this issue in the SST task). Therefore, the disparity in findings across the reviewed literature may be partly related to differences in how emotional content is incorporated into inhibition paradigms, and even in how its relevance to the task is defined. Importantly, here, we consider stimuli as emotionally task-relevant if their emotional content serves as an explicit criterion for task completion, whether the emotion is included in the go stimulus, the no-go stimulus, or both (emotional influences prior to the go stimulus are not considered).

For our fifth objective, we explored additional factors that may influence the emotional modulation of response inhibition, including the type of inhibition task and the nature of the emotional stimuli used. Regarding stimulus type, findings suggest that emotional effects on response inhibition are consistently observed across different emotional stimuli, with effects reported in 71% of studies using words, 67% using scenes, and 65% using faces. These results suggest that despite the notable differences among these types of emotional stimuli (Yuan et al., 2019), their influence on response inhibition at the behavioral level remains comparable. It is also worth noting that sound was rarely used as a stimulus. Further research is needed to determine whether emotional sounds have a distinct effect on response inhibition, given that their processing pathways differ from those of the more commonly used visual stimuli. Moreover, an important factor beyond stimulus characteristics was the type of task in which the stimuli were embedded. We found that any kind of emotional effects on behavior were more frequently observed in studies using the SST (approximately 80%) compared to those using the GNG task (around 50%) or the overall trend of the review (about 60% of all studies). This discrepancy is likely due to two task-related factors rather than differences in the stimuli themselves. First, the SST is considered a more pure measure of response inhibition, as it allows for a more detailed analysis of the stopping process and involves fewer interfering cognitive processes than the GNG (Aron, 2011; Congdon et al., 2012). Consequently, emotional effects on response inhibition are more likely to be detected. Second, a substantial number of studies using the GNG task employed a design in which No-Go stimuli appeared with relatively high frequency (50% of trials), reducing response prepotency and lowering inhibitory demands. As a result, detecting emotional effects on response inhibition under such conditions may be less likely.

The exploratory analysis of the neural correlates of emotional modulation in response inhibition also revealed mixed and inconclusive evidence. Notably, ERP and fMRI techniques were more likely to detect effects compared to behavioral measures, with some studies reporting neural-level differences even in the absence of observable behavioral changes. This suggests that these methods are more sensitive than behavioral analyses alone. Overall, emotional response inhibition has been shown to engage the same brain regions typically involved in inhibition with neutral stimuli (e.g., dorsal striatum and lateral prefrontal cortex), along with additional regions such as the ventral striatum, orbitofrontal cortex, and anterior cingulate cortex (Albert et al., 2010, 2012; Goldstein et al., 2007; Zhuang et al., 2021). Another key observation is that effects were sometimes detected in N2 but not in P3 components (or vice versa), and in some cases, opposite effects were found between N2 and P3. This highlights the fact that these two indices of inhibition may not be equally informative. Indeed, previous research suggests that while the P3 component is a more direct reflection of response inhibition processes, the N2 component is more closely related to conflict monitoring and novelty detection (Albert et al., 2013). However, even when distinguishing between N2 and P3, the results remained inconsistent.

An overview of the results obtained in this review suggests that no single factor related to emotional stimuli consistently drives behavioral effects on response inhibition. Given the mixed findings in the literature, the answer likely lies in how these variables combine and interact with each other, as well as with other potential influencing factors (Schindler and Bublatzky, 2020; Yuan et al., 2019). For instance, research on other forms of cognitive control -measured using tasks like Stroop and Flanker- has shown that negative stimuli performance varies depending on resource availability, attentional factors, and concurrent top-down processes (Cohen and Henik, 2012). A similar pattern may occur in response inhibition, where effects could be more evident in specific subgroups of studies formed based on a combination of variables. While exploring these effects within subgroups would be valuable, this was not feasible here due to the limited information available on these factors in the reviewed studies and the small sample sizes of subgroups formed by combining our variables of interest. In the few instances where sample sizes were large enough to draw conclusions, the results aligned with the general trends observed. For example, when trying to discern effects specific to one task or the other, the number of SST studies where valence or arousal emerged as a clear driver of effects was too low to draw any conclusions, but positive valence was found more often associated with less efficient response inhibition when we analyzed the results separately for studies using only a GNG task (which coincides with the general trend of the review). Other additional factors of interest emerging from the reviewed literature include the sample's underlying traits, such as sex/gender distribution, age, and psychological profiles (see Supplementary Table 1 for an overview). However, due to the limited number of studies explicitly considering these variables, we are unable to draw conclusions at this time. Furthermore, it would also be of interest to ascertain the impact of other factors outside this review, such as varying levels of competition for available cognitive resources (Pessoa, 2009), as previously mentioned.

Moreover, it should be noted that the SSRT is a latent variable primarily estimated using the horse-race model. While this model has been crucial in advancing the field, violations of its assumptions can result in inaccurate or even erroneous SSRT estimates (Bissett et al., 2021). Furthermore, failures in initiating the inhibition process (commonly referred to as “stop trigger failures”) may further compromise the accuracy of SSRT estimations (Matzke et al., 2017). Specifically, research has shown that trigger failures can lead to substantial overestimation of SSRTs (Band et al., 2003). Given these methodological concerns, the conclusions drawn from the emotional SST studies included here should be interpreted with caution. On a related note, the behavioral correlates of response inhibition involve multiple cognitive processes, some of which occur before the actual implementation of response inhibition. Consequently, differences in SSRT -and also in commission errors- observed across emotional conditions (e.g., positive vs. negative valence) may arise not only from the influence of emotional content of stimuli on the inhibition process itself, but also from emotional modulation of earlier cognitive processes (Doekemeijer et al., 2021; Verbruggen et al., 2014; Yiend, 2010). For instance, the probability of trigger failures, often associated with attentional lapses, may vary depending on the emotional content of the stimuli (e.g., their valence or arousal), thereby influencing the SSRT linked to each emotional condition. Therefore, it is important to explore whether the emotional modulation of the behavioral correlates of inhibition affects the inhibition itself and/or preceding cognitive processes. The use of brain activity measures (particularly electromagnetic ones due to their high temporal resolution) and new models of action-stopping (Bissett and Poldrack, 2022), could be particularly useful in shedding light on this issue.

Notably, most studies on the emotional modulation of response inhibition have focused on global and reactive (stimulus-driven) inhibition. However, inhibitory control can also take more complex forms, such as proactive inhibition (anticipating and preparing to suppress an upcoming response) and selective inhibition (suppressing certain responses but not others, or inhibiting reactions to specific stimuli while continuing to respond to others), as described by Aron (2011). Further research is therefore needed to determine whether emotional stimuli exert distinct effects on other forms of response inhibition beyond global reactive inhibition. In this regard, some evidence suggests that affective modulation may differ at least between reactive and proactive response inhibition (Xu et al., 2016b). Additionally, it is necessary to explore whether emotion can modulate reactive inhibition through its influence on proactive inhibition (and vice versa). The studies reviewed here employ tasks aimed at examining reactive inhibition, but they may require varying degrees of proactive control (Meyer and Bucci, 2016), depending on details that are not always explicitly reported such as the particularities of the design of the task and the instructions provided to participants (Verbruggen and Logan, 2009). Additionally, it seems necessary to explore the affective modulation of response inhibition in real-world settings beyond the laboratory (Hannah and Aron, 2021). Findings from controlled experiments may not be fully generalizable to natural environments and everyday situations.

In light of the above, this systematic review may provide valuable insights for future research on the influence of emotional stimuli on response inhibition: (1) It is crucial to control both the valence and arousal levels of emotional stimuli, regardless of their type (e.g., faces, pictures, words or sounds). Studies should be designed to examine the effects of valence and arousal both independently and in interaction; (2) Researchers are encouraged to use stimuli from large, standardized, and recently published affective databases that align with the sociocultural and demographic characteristics of the study's participants. Moreover, obtaining valence and arousal ratings directly from the experimental sample can help confirm whether stimuli are perceived as intended and provide valuable data for linking the participants' own subjective evaluation of emotional stimuli to behavioral and neural measures; (3) The discrete model of emotions may offer additional insights into emotional modulation of response inhibition beyond what is revealed by the two-dimensional circumplex model; (4) The field would benefit from future research that examines the interactions between all the factors discussed here, rather than studying each one in isolation. Additionally, it is important to consider the characteristics and methodological challenges of the inhibition tasks (including the SSRT estimation), and the task relevance of the emotional aspects of the stimuli—ensuring a clear definition of task relevance in each study, as outlined above; (5) Other factors, outside those we focused on in this review, may also be of interest and should be studied further, both independently and in relation to the other factors. These include the cognitive load of the inhibition task, as well as the underlying characteristics of the samples used; (6) Finally, research should go beyond global reactive inhibition to explore other forms of inhibitory control, such as proactive and selective inhibition, which may be particularly relevant in real-world settings.

In sum, this systematic review suggests that emotional stimuli modulate response inhibition in adult non-clinical populations, but the underlying mechanisms remain uncertain. Although valence appears to have a greater influence than arousal, the evidence remains inconsistent. The type of inhibition task (SST) and the relevance of emotional stimuli to task goals (task-relevant) also appear to be potential factors in facilitating the emotional modulation of response inhibition. However, the behavioral effects of emotional stimuli on response inhibition are likely influenced by a complex interplay of multiple factors, with no single factor standing out, suggesting that future research should explore how these factors interact.

## References

[B1] Agudelo-Orjuela P. de Vega M. Beltrán D. (2021). Mutual influence between emotional language and inhibitory control processes. Evidence from an event-related potential study. Psychophysiology 58:e13743. 10.1111/psyp.13743 33278304

[B2] Albert J. López-Martín S. Arza R. Palomares N. Hoyos S. Carretié L. . (2019). Response inhibition in borderline personality disorder: neural and behavioral correlates. Biol. Psychol. 143, 32–40. 10.1016/j.biopsycho.2019.02.003 30772405

[B3] Albert J. López-Martín S. Carretié L. (2010). Emotional context modulates response inhibition: neural and behavioral data. Neuroimage 49, 914–921. 10.1016/j.neuroimage.2009.08.045 19716425

[B4] Albert J. López-Martín S. Hinojosa J. A. Carretié L. (2013). Spatiotemporal characterization of response inhibition. NeuroImage 76, 272–281. 10.1016/j.neuroimage.2013.03.011 23523776

[B5] Albert J. López-Martín, S. Tapia M. Montoya D. Carretie L. (2012). The role of the anterior cingulate cortex in emotional response inhibition. Hum. Brain Mapp. 33, 2147–2160. 10.1002/hbm.21347 21901794 PMC6870140

[B6] Allen K. J. Hooley J. M. (2019). Negative emotional action termination (NEAT): support for a cognitive mechanism underlying negative urgency in nonsuicidal self-injury. Behav. Ther. 50, 924–937. 10.1016/j.beth.2019.02.001 31422848

[B7] Amin Z. Epperson C. N. Constable R. T. Canli T. (2006). Effects of estrogen variation on neural correlates of emotional response inhibition. NeuroImage 32, 457–464. 10.1016/j.neuroimage.2006.03.013 16644236

[B8] Andreu C. I. Palacios I. Moënne-Loccoz C. López V. Franken I. H. Cosmelli D. . (2019). Enhanced response inhibition and reduced midfrontal theta activity in experienced Vipassana meditators. Sci. Rep. 9:13215. 10.1038/s41598-019-49714-9 31519984 PMC6744491

[B9] Aron A. R. (2011). From reactive to proactive and selective control: developing a richer model for stopping inappropriate responses. Biol. Psychiatry 69, e55–68. 10.1016/j.biopsych.2010.07.024 20932513 PMC3039712

[B10] Asci O. Braem S. Park H. R. Boehler C. N. Krebs R. M. (2019). Neural correlates of reward-related response tendencies in an equiprobable Go/NoGo task. Cognit. Affect. Behav. Neurosci. 19, 555–567. 10.3758/s13415-019-00692-5 30788804

[B11] Atkinson-Clement C. Porte C. A. de Liege A. Wattiez N. Klein Y. Beranger Y. . (2020). Neural correlates and role of medication in reactive motor impulsivity in Tourette disorder. Cortex 125, 60–72. 10.1016/j.cortex.2019.12.007 31978743

[B12] Aziz-Safaie T. Müller V. I. Langner R. Eickhoff S. B. Cieslik E. C. (2024). The effect of task complexity on the neural network for response inhibition: an ale meta-analysis. Neurosci. Biobehav. Rev. 158:105544. 10.1016/j.neubiorev.2024.105544 38220034 PMC11130604

[B13] Bailen N. H. Green L. M. Thompson R. J. (2019). Understanding emotion in adolescents: a review of emotional frequency, intensity, instability, and clarity. Emot. Rev. 11, 63–73. 10.1177/1754073918768878

[B14] Band G. P. Van Der Molen M. W. Logan G. D. (2003). Horse-race model simulations of the stop-signal procedure. Acta Psychol. 112, 105–142. 10.1016/S0001-6918(02)00079-3 12521663

[B15] Bari A. Robbins T. W. (2013). Inhibition and impulsivity: behavioral and neural basis of response control. Prog. Neurobiol. 108, 44–79. 10.1016/j.pneurobio.2013.06.005 23856628

[B16] Battaglia S. Cardellicchio P. Di Fazio C. Nazzi C. Fracasso A. Borgomaneri S. (2022a). The influence of vicarious fear-learning in “infecting” reactive action inhibition. Front. Behav. Neurosci. 16:946263. 10.3389/fnbeh.2022.946263 35941933 PMC9355887

[B17] Battaglia S. Cardellicchio P. Di Fazio C. Nazzi C. Fracasso A. Borgomaneri S. (2022b). Stopping in (e) motion: reactive action inhibition when facing valence-independent emotional stimuli. Front. Behav. Neurosci. 16:998714. 10.3389/fnbeh.2022.998714 36248028 PMC9561776

[B18] Battaglia S. Serio G. Scarpazza C. D'Ausilio A. Borgomaneri S. (2021). Frozen in (e) motion: how reactive motor inhibition is influenced by the emotional content of stimuli in healthy and psychiatric populations. Behav. Res. Ther. 146:103963. 10.1016/j.brat.2021.103963 34530318

[B19] Beatty G. F. Cranley N. M. Carnaby G. Janelle C. M. (2016). Emotions predictably modify response times in the initiation of human motor actions: a meta-analytic review. Emotion 16:237. 10.1037/emo0000115 26461243

[B20] Benvenuti S. M. Buodo G. Palomba D. (2017). Appetitive and aversive motivation in dysphoria: a time-domain and time-frequency study of response inhibition. Biol. Psychol. 125, 12–27. 10.1016/j.biopsycho.2017.02.007 28238885

[B21] Benvenuti S. M. Sarlo M. Buodo G. Mento G. Palomba D. (2015). Influence of impulsiveness on emotional modulation of response inhibition: an ERP study. Clin. Neurophysiol. 126, 1915–1925. 10.1016/j.clinph.2014.12.012 25595704

[B22] Berlin H. A. Schulz K. P. Zhang S. Turetzky R. Rosenthal D. Goodman W. . (2015). Neural correlates of emotional response inhibition in obsessive-compulsive disorder: a preliminary study. Psychiatry Res. Neuroimaging 234, 259–264. 10.1016/j.pscychresns.2015.09.019 26456416

[B23] Bissett P. G. Jones H. M. Poldrack R. A. Logan G. D. (2021). Severe violations of independence in response inhibition tasks. Sci. Adv. 7:*eabf* 4355. 10.1126/sciadv.abf4355 33731357 PMC7968836

[B24] Bissett P. G. Poldrack R. A. (2022). Estimating the time to do nothing: toward next-generation models of response inhibition. Curr. Direct. Psychol. Sci. 31, 556–563. 10.1177/09637214221121753

[B25] Bramer W. M. Rethlefsen M. L. Kleijnen J. Franco O. H. (2017). Optimal database combinations for literature searches in systematic reviews: a prospective exploratory study. Syst. Rev. 6:245. 10.1186/s13643-017-0644-y 29208034 PMC5718002

[B26] Brosch T. Pourtois G. Sander D. (2010). The perception and categorisation of emotional stimuli: a review. Cognit. Emot. 24, 377–400. 10.1080/02699930902975754

[B27] Brown M. R. Benoit J. R. Juhás M. Lebel R. M. MacKay M. Dametto E. . (2015). Neural correlates of high-risk behavior tendencies and impulsivity in an emotional Go/NoGo fMRI task. Front. Syst. Neurosci. 9:24. 10.3389/fnsys.2015.00024 25805975 PMC4354310

[B28] Brown M. R. Lebel R. M. Dolcos F. Wilman A. H. Silverstone P. H. Pazderka H. . (2012). Effects of emotional context on impulse control. NeuroImage 63, 434–446. 10.1016/j.neuroimage.2012.06.056 22781161

[B29] Buodo G. Sarlo M. Mento G. Messerotti Benvenuti S. Palomba D. (2017). Unpleasant stimuli differentially modulate inhibitory processes in an emotional Go/NoGo task: an event-related potential study. Cognit. Emot. 31, 127–138. 10.1080/02699931.2015.1089842 26403599

[B30] Calbi M. Montalti M. Pederzani C. Arcuri E. Umiltà M. A. Gallese V. . (2022). Emotional body postures affect inhibitory control only when task-relevant. Front. Psychol. 13:1035328. 10.3389/fpsyg.2022.1035328 36405118 PMC9669573

[B31] Camfield D. A. Burton T. K. De Blasio F. M. Barry R. J. Croft R. J. (2018). ERP components associated with an indirect emotional stop signal task in healthy and depressed participants. Int. J. Psychophysiol. 124, 12–25. 10.1016/j.ijpsycho.2017.12.008 29278691

[B32] Carretié L. (2014). Exogenous (automatic) attention to emotional stimuli: a review. Cognit. Affect. Behav. Neurosci. 14, 1228–1258. 10.3758/s13415-014-0270-2 24683062 PMC4218981

[B33] Chen W. de Hemptinne C. Miller A. M. Leibbrand M. Little S. J. Lim D. A. . (2020). Prefrontal-subthalamic hyperdirect pathway modulates movement inhibition in humans. Neuron 106, 579–588. 10.1016/j.neuron.2020.02.012 32155442 PMC7274135

[B34] Chester D. S. Lynam D. R. Milich R. Powell D. K. Andersen A. H. DeWall C. N. . (2016). How do negative emotions impair self-control? A neural model of negative urgency. NeuroImage 132, 43–50. 10.1016/j.neuroimage.2016.02.024 26892861 PMC4851933

[B35] Chiu P. H. Holmes A. J. Pizzagalli D. A. (2008). Dissociable recruitment of rostral anterior cingulate and inferior frontal cortex in emotional response inhibition. NeuroImage 42, 988–997. 10.1016/j.neuroimage.2008.04.248 18556218 PMC2604817

[B36] Cohen N. Henik A. (2012). Do irrelevant emotional stimuli impair or improve executive control? Front. Integr. Neurosci. 6:33. 10.3389/fnint.2012.00033 22719722 PMC3376948

[B37] Cohen-Gilbert J. E. Thomas K. M. (2013). Inhibitory control during emotional distraction across adolescence and early adulthood. Child Dev. 84, 1954–1966. 10.1111/cdev.12085 23506340 PMC3688699

[B38] Congdon E. Mumford J. A. Cohen J. R. Galvan A. Canli T. Poldrack R. A. . (2012). Measurement and reliability of response inhibition. Front. Psychol. 3:37. 10.3389/fpsyg.2012.00037 22363308 PMC3283117

[B39] Contreras D. Megías A. Maldonado A. Cándido A. Catena A. (2013). Facilitation and interference of behavioral responses by task-irrelevant affect-laden stimuli. Motivat. Emot. 37, 496–507. 10.1007/s11031-012-9327-0

[B40] Cromheeke S. Mueller S. C. (2014). Probing emotional influences on cognitive control: an ALE meta-analysis of cognition emotion interactions. Brain Struct. Function 219, 995–1008. 10.1007/s00429-013-0549-z 23563751

[B41] Crosbie J. Arnold P. Paterson A. Swanson J. Dupuis A. Li X. . (2013). Response inhibition and ADHD traits: correlates and heritability in a community sample. J. Abnorm. Child Psychol. 41, 497–507. 10.1007/s10802-012-9693-9 23315233 PMC3600128

[B42] De Houwer J. Tibboel H. (2010). Stop what you are not doing! Emotional pictures interfere with the task not to respond. Psychon. Bull. Rev. 17, 699–703. 10.3758/PBR.17.5.699 21037169

[B43] De Sanctis P. Foxe J. J. Czobor P. Wylie G. R. Kamiel S. M. Huening J. . (2013). Early sensory-perceptual processing deficits for affectively valenced inputs are more pronounced in schizophrenia patients with a history of violence than in their non-violent peers. Soc. Cognit. Affect. Neurosci. 8, 678–687. 10.1093/scan/nss052 22563006 PMC3739916

[B44] Demers L. A. Hunt R. H. Cicchetti D. Cohen-Gilbert J. E. Rogosch F. A. Toth S. L. . (2022). Impact of childhood maltreatment and resilience on behavioral and neural patterns of inhibitory control during emotional distraction. Dev. Psychopathol. 34, 1260–1271. 10.1017/S0954579421000055 33827733 PMC8497637

[B45] Diamond A. (2013). Executive functions. Annu. Rev. Psychol. 64, 135–168. 10.1146/annurev-psych-113011-143750 23020641 PMC4084861

[B46] Ding J. Wang Y. Wang C. d'Oleire Uquillas, F. He Q. Cheng L, Zou, Z. (2020). Negative impact of sadness on response inhibition in females: an explicit emotional stop signal task fMRI study. Front. Behav. Neurosci. 14:119. 10.3389/fnbeh.2020.00119 32903296 PMC7396530

[B47] Doekemeijer R. A. Verbruggen F. Boehler C. N. (2021). Face the (trigger) failure: trigger failures strongly drive the effect of reward on response inhibition. Cortex 139, 166–177. 10.1016/j.cortex.2021.02.025 33873037

[B48] Downes M. J. Brennan M. L. Williams H. C. Dean R. S. (2016). Development of a critical appraisal tool to assess the quality of cross-sectional studies (AXIS). BMJ Open 6:*e*011458. 10.1136/bmjopen-2016-011458 27932337 PMC5168618

[B49] Eder A. B. Hommel B. (2013). Anticipatory control of approach and avoidance: an ideomotor approach. Emot. Rev. 5, 275–279. 10.1177/1754073913477505

[B50] Ekman P. (1992). An argument for basic emotions. Cognit. Emot. 6, 169–200. 10.1080/02699939208411068

[B51] Fink-Lamotte J. Widmann A. Sering K. Schröger E. Exner C. (2021). Attentional processing of disgust and fear and its relationship with contamination-based obsessive-compulsive symptoms: stronger response urgency to disgusting stimuli in disgust-prone individuals. Front. Psychiatry 12:596557. 10.3389/fpsyt.2021.596557 34163378 PMC8215551

[B52] Fournier L. F. McDonald J. B. Clayson P. E. Verona E. (2021). Psychopathic traits, inhibition, and positive and negative emotion: results from an emotional Go/No-Go task. Psychophysiology 58:e13815. 10.1111/psyp.13815 33768574 PMC8169549

[B53] García-Blanco A. C. Perea M. Livianos L. (2013). Mood-congruent bias and attention shifts in the different episodes of bipolar disorder. Cognit. Emot. 27, 1114–1121. 10.1080/02699931.2013.764281 23360445

[B54] Goldstein M. Brendel G. Tuescher O. Pan H. Epstein J. Beutel M. . (2007). Neural substrates of the interaction of emotional stimulus processing and motor inhibitory control: an emotional linguistic go/no-go fMRI study. Neuroimage 36, 1026–1040. 10.1016/j.neuroimage.2007.01.056 17509899

[B55] Gole M. Köchel A. Schäfer A. Schienle A. (2012). Threat engagement, disengagement, and sensitivity bias in worry-prone individuals as measured by an emotional go/no-go task. J. Behav. Ther. Exp. Psychiatry 43, 532–539. 10.1016/j.jbtep.2011.07.002 21819812

[B56] Greif T. R. Waring J. D. (2018). Emotional contrast and psychological function impact response inhibition to threatening faces. Motivat. Emot. 42, 920–930. 10.1007/s11031-018-9709-z 30581242 PMC6301040

[B57] Gupta R. Singh J. P. (2021). Only irrelevant angry, but not happy, expressions facilitate the response inhibition. Attent. Percept. Psychophys. 83, 114–121. 10.3758/s13414-020-02186-w 33146816

[B58] Gupta R. Singh J. P. (2023). Irrelevant emotional expressions interfered with response inhibition: the role of contrast emotions. J. Cognit. Psychol. 35, 677–687. 10.1080/20445911.2023.2242101

[B59] Haddaway N. R. Page M. J. Pritchard C. C. McGuinness L. A. (2022). PRISMA2020: an R package and Shiny app for producing PRISMA 2020-compliant flow diagrams, with interactivity for optimised digital transparency and open synthesis. Campbell Syst. Rev. 18:*e*1230. 10.1002/cl2.1230 36911350 PMC8958186

[B60] Hannah R. Aron A. R. (2021). Towards real-world generalizability of a circuit for action-stopping. Nat. Rev. Neurosci. 22, 538–552. 10.1038/s41583-021-00485-1 34326532 PMC8972073

[B61] Hare T. A. Tottenham N. Davidson M. C. Glover G. H. Casey B. J. (2005). Contributions of amygdala and striatal activity in emotion regulation. Biol. Psychiatry 57, 624–632. 10.1016/j.biopsych.2004.12.038 15780849

[B62] Harlé K. M. Shenoy P. Paulus M. P. (2013). The influence of emotions on cognitive control: feelings and beliefs-where do they meet? Front. Hum. Neurosci. 7:508. 10.3389/fnhum.2013.00508 24065901 PMC3776943

[B63] Hervault M. Soh C. Wessel J. R. (2025). Does the stop-signal P3 reflect inhibitory control? Cortex 183, 232–250. 10.1016/j.cortex.2024.12.005 39754857 PMC11839379

[B64] Hinojosa J. A. Moreno E. M. Ferré P. (2020). Affective neurolinguistics: towards a framework for reconciling language and emotion. Lang. Cognit. Neurosci. 35, 813–839. 10.1080/23273798.2019.1620957

[B65] Jia L. X. Zheng Q. Cui J. F. Shi H. S. Ye J. Y. Yang T. X. . (2023). Proactive and reactive response inhibition of individuals with high schizotypy viewing different facial expressions: an ERP study using an emotional stop-signal task. Brain Res. 1799:148191. 10.1016/j.brainres.2022.148191 36463955

[B66] Jones A. Field M. (2015). Alcohol-related and negatively valenced cues increase motor and oculomotor disinhibition in social drinkers. Exp. Clin. Psychopharmacol. 23, 122–129. 10.1037/pha0000011 25730418 PMC4386809

[B67] Kakuszi B. Szuromi B. Bitter I. Czobor P. (2020). Attention deficit hyperactivity disorder: last in, first out-delayed brain maturation with an accelerated decline? Eur. Neuropsychopharmacol. 34, 65–75. 10.1016/j.euroneuro.2020.03.011 32279924

[B68] Kalanthroff E. Cohen N. Henik A. (2013). Stop feeling: inhibition of emotional interference following stop-signal trials. Front. Hum. Neurosci. 7:78. 10.3389/fnhum.2013.00078 23503817 PMC3596782

[B69] Kampa M. Sebastian A. Tüscher O. Stark R. Klucken T. (2023). Refocus on stopping! Replication of reduced right amygdala reactivity to negative, visual primes during inhibition of motor responses. NeuroImage Rep. 3:100151. 10.1016/j.ynirp.2022.100151 PMC12172732 40568047

[B70] Krypotos A. M. Jahfari S. van Ast V. A. Kindt M. Forstmann B. U. (2011). Individual differences in heart rate variability predict the degree of slowing during response inhibition and initiation in the presence of emotional stimuli. Front. Psychol. 2:12698. 10.3389/fpsyg.2011.00278 22059080 PMC3204574

[B71] Littman R. Takács Á. (2017). Do all inhibitions act alike? A study of go/no-go and stop-signal paradigms. PLoS ONE 12:e0186774. 10.1371/journal.pone.0186774 29065184 PMC5655479

[B72] Liu C. Dai J. Chen Y. Qi Z. Xin F. Zhuang Q. . (2021). Disorder-and emotional context-specific neurofunctional alterations during inhibitory control in generalized anxiety and major depressive disorder. NeuroImage Clin. 30:102661. 10.1016/j.nicl.2021.102661 33866301 PMC8060548

[B73] Liu T. Xiao T. Shi J. (2018). Neural correlates of response inhibition and conflict control on facial expressions. Front. Hum. Neurosci. 11:657. 10.3389/fnhum.2017.00657 29375351 PMC5767249

[B74] Lodha S. Gupta R. (2024). Irrelevant angry, but not happy, faces facilitate response inhibition in mindfulness meditators. Curr. Psychology, 43, 811–826. 10.1007/s12144-023-04384-9

[B75] Ma Z. Li J. Niu Y. Yu J. Yang L. (2013). Age differences in emotion recognition between Chinese younger and older adults. Psychol. Record. 63, 629–640. 10.11133/j.tpr.2013.63.3.015

[B76] Mallorquí-Bagué N. Testa G. Lozano-Madrid M. Vintró-Alcaraz C. Sánchez I. Riesco N. . (2020). Emotional and non-emotional facets of impulsivity in eating disorders: from anorexia nervosa to bulimic spectrum disorders. Eur. Eat. Disord. Rev. 28, 410–422. 10.1002/erv.2734 32212204

[B77] Mancini C. Falciati L. Maioli C. Mirabella G. (2022). Happy facial expressions impair inhibitory control with respect to fearful facial expressions but only when task-relevant. Emotion 22, 142–152. 10.1037/emo0001058 34968143

[B78] Mar K. Townes P. Pechlivanoglou P. Arnold P. Schachar R. (2022). Obsessive compulsive disorder and response inhibition: meta-analysis of the stop-signal task. J. Psychopathol. Clin. Sci. 131, 152–161. 10.1037/abn0000732 34968087

[B79] Matzke D. Love J. Heathcote A. (2017). A Bayesian approach for estimating the probability of trigger failures in the stop-signal paradigm. Behav. Res. Methods 49, 267–281. 10.3758/s13428-015-0695-8 26822670 PMC5352806

[B80] Mennella R. Sarlo M. Messerotti Benvenuti S. Buodo G. Mento G. Palomba D. . (2017). The two faces of avoidance: time-frequency correlates of motivational disposition in blood phobia. Psychophysiology 54, 1606–1620. 10.1111/psyp.12904 28580599

[B81] Menzies L. Achard S. Chamberlain S. R. Fineberg N. Chen C. H. Del Campo N. . (2007). Neurocognitive endophenotypes of obsessive-compulsive disorder. Brain 130, 3223–3236. 10.1093/brain/awm205 17855376

[B82] Meyer H. C. Bucci D. J. (2016). Neural and behavioral mechanisms of proactive and reactive inhibition. Learn. Memory 23, 504–514. 10.1101/lm.040501.115 27634142 PMC5026209

[B83] Mirabella G. Grassi M. Bernardis P. (2024). The role of task relevance in saccadic responses to facial expressions. Ann. N. Y. Acad. Sci. 1540, 324–337. 10.1111/nyas.15221 39316839

[B84] Mirabella G. Grassi M. Mezzarobba S. Bernardis P. (2023). Angry and happy expressions affect forward gait initiation only when task relevant. Emotion 23, 387–399. 10.1037/emo0001112 35588387

[B85] Montalti M. Mirabella G. (2023). Unveiling the influence of task-relevance of emotional faces on behavioral reactions in a multi-face context using a novel Flanker-Go/No-go task. Sci. Rep. 13:20183. 10.1038/s41598-023-47385-1 37978229 PMC10656465

[B86] Moors A. Fischer M. (2019). Demystifying the role of emotion in behaviour: toward a goal-directed account. Cognit. Emot. 33, 94–100. 10.1080/02699931.2018.1510381 30102113

[B87] Moretta T. Buodo G. (2021). Response inhibition in problematic social network sites use: an ERP study. Cognit. Affect. Behav. Neurosci. 21, 868–880. 10.3758/s13415-021-00879-9 33674995 PMC8354934

[B88] Morie K. P. Garavan H. Bell R. P. De Sanctis P. Krakowski M. I. Foxe J. J. . (2014). Intact inhibitory control processes in abstinent drug abusers (II): a high-density electrical mapping study in former cocaine and heroin addicts. Neuropharmacology 82, 151–160. 10.1016/j.neuropharm.2013.02.023 23507565

[B89] Moses M. Tiego J. Demontis D. Bragi Walters G. Stefansson H. Stefansson K. . (2022). Working memory and reaction time variability mediate the relationship between polygenic risk and ADHD traits in a general population sample. Mole. Psychiatry, 27, 5028–5037. 10.1038/s41380-022-01775-5 36151456 PMC9763105

[B90] Mulckhuyse M. (2018). The influence of emotional stimuli on the oculomotor system: a review of the literature. Cognit. Affect. Behav. Neurosci. 18, 411–425. 10.3758/s13415-018-0590-8 29633198

[B91] Narayanan N. S. Wessel J. R. Greenlee J. D. (2020). The fastest way to stop: inhibitory control and IFG-STN hyperdirect connectivity. Neuron 106, 549–551. 10.1016/j.neuron.2020.04.017 32437650 PMC8188636

[B92] Page M. J. McKenzie J. E. Bossuyt P. M. Boutron I. Hoffmann T. C. Mulrow C. D. . (2021). The PRISMA 2020 statement: an updated guideline for reporting systematic reviews. BMJ 372:n71. 10.31222/osf.io/v7gm2 33782057 PMC8005924

[B93] Pandey S. Gupta R. (2022). Irrelevant positive emotional information facilitates response inhibition only under a high perceptual load. Sci. Rep. 12:14591. 10.1038/s41598-022-17736-5 36028535 PMC9418248

[B94] Panksepp J. Watt D. (2011). What is basic about basic emotions? Lasting lessons from affective neuroscience. Emot. Rev. 3, 387–396. 10.1177/1754073911410741

[B95] Pessoa L. (2009). How do emotion and motivation direct executive control? Trends Cognit. Sci. 13, 160–166. 10.1016/j.tics.2009.01.006 19285913 PMC2773442

[B96] Pessoa L. Padmala S. Kenzer A. Bauer A. (2012). Interactions between cognition and emotion during response inhibition. Emotion 12:192. 10.1037/a0024109 21787074 PMC3208031

[B97] Pool E. Brosch T. Delplanque S. Sander D. (2016). Attentional bias for positive emotional stimuli: a meta-analytic investigation. Psycholo. Bull. 142:79. 10.1037/bul0000026 26390266

[B98] Ramos R. Vaz A. R. Rodrigues T. F. Baenas I. Fernández-Aranda F. Machado P. P. (2024). Exploring the relationship between emotion regulation, inhibitory control, and eating psychopathology in a non-clinical sample. Eur. Eat. Disord. Rev. 32, 66–79. 10.1002/erv.3024 37581422

[B99] Ramos-Loyo J. Angulo-Chavira A. Llamas-Alonso L. A. González-Garrido A. A. (2016). Sex differences in emotional contexts modulation on response inhibition. Neuropsychologia 91, 290–298. 10.1016/j.neuropsychologia.2016.08.023 27565638

[B100] Ramos-Loyo J. Juárez-García C. Llamas-Alonso L. A. Angulo-Chavira A. Q. Romo-Vázquez R. Vélez-Pérez H. . (2021). Inhibitory control under emotional contexts in women with borderline personality disorder: an electrophysiological study. J. Psychiat. Res. 132, 182–190. 10.1016/j.jpsychires.2020.10.014 33132135

[B101] Raud L. Westerhausen R. Dooley N. Huster R. J. (2020). Differences in unity: the go/no-go and stop signal tasks rely on different mechanisms. NeuroImage 210:116582. 10.1016/j.neuroimage.2020.116582 31987997

[B102] Reitsema A. M. Jeronimus B. F. van Dijk M. de Jonge P. (2022). Emotion dynamics in children and adolescents: a meta-analytic and descriptive review. Emotion 22, 374–396. 10.1037/emo0000970 34843305

[B103] Ruffman T. Henry J. D. Livingstone V. Phillips L. H. (2008). A meta-analytic review of emotion recognition and aging: implications for neuropsychological models of aging. Neurosci. Biobehav. Rev. 32, 863–881. 10.1016/j.neubiorev.2008.01.001 18276008

[B104] Russell J. A. (1980). A circumplex model of affect. J. Pers. Soc. Psychol. 39:1161. 10.1037/h0077714

[B105] Russell J. A. (2003). Core affect and the psychological construction of emotion. Psychol. Rev. 110, 145–172. 10.1037/0033-295X.110.1.145 12529060

[B106] Sakaki M. Niki K. Mather M. (2012). Beyond arousal and valence: the importance of the biological versus social relevance of emotional stimuli. Cognit. Affect. Behav. Neurosci. 12, 115–139. 10.3758/s13415-011-0062-x 21964552 PMC3306241

[B107] Sánchez-Carmona A. J. Albert J. Hinojosa J. A. (2016). Neural and behavioral correlates of selective stopping: evidence for a different strategy adoption. Neuroimage 139, 279–293. 10.1016/j.neuroimage.2016.06.043 27355436

[B108] Sánchez-Carmona A. J. Santaniello G. Capilla A. Hinojosa J. A. Albert J. (2019). Oscillatory brain mechanisms supporting response cancellation in selective stopping strategies. Neuroimage 197, 295–305. 10.1016/j.neuroimage.2019.04.066 31034967

[B109] Schachar R. Logan G. D. Robaey P. Chen S. Ickowicz A. Barr C. . (2007). Restraint and cancellation: multiple inhibition deficits in attention deficit hyperactivity disorder. J. Abnorm. Child Psychol. 35, 229–238. 10.1007/s10802-006-9075-2 17351752

[B110] Schall J. D. Palmeri T. J. Logan G. D. (2017). Models of inhibitory control. Phil. Trans. R. Soc. B. Biol. Sci. 372:20160193. 10.1098/rstb.2016.0193 28242727 PMC5332852

[B111] Schel M. A. Crone E. A. (2013). Development of response inhibition in the context of relevant versus irrelevant emotions. Front. Psychol. 4:383. 10.3389/fpsyg.2013.00383 23847560 PMC3698449

[B112] Schindler S. Bublatzky F. (2020). Attention and emotion: an integrative review of emotional face processing as a function of attention. Cortex 130, 362–386. 10.1016/j.cortex.2020.06.010 32745728

[B113] Schmaußer M. Laborde S. (2023). Tonic and phasic cardiac vagal activity predict cognitive-affective processing in an emotional stop-signal task. Int. J. Psychophysiol. 191, 9–18. 10.1016/j.ijpsycho.2023.06.008 37355042

[B114] Senderecka M. (2016). Threatening visual stimuli influence response inhibition and error monitoring: an event-related potential study. Biol. Psychol. 113, 24–36. 10.1016/j.biopsycho.2015.11.003 26599814

[B115] Senderecka M. (2018). Emotional enhancement of error detection-the role of perceptual processing and inhibition monitoring in failed auditory stop trials. Cognit. Affect. Behav. Neurosci. 18, 1–20. 10.3758/s13415-017-0546-4 29076064 PMC5823965

[B116] Senderecka M. Ociepka M. Matyjek M. Kroczek B. (2018). Post-error brain activity correlates with incidental memory for negative words. Front. Hum. Neurosci. 12:178. 10.3389/fnhum.2018.00178 29867408 PMC5951961

[B117] Shafer A. T. Matveychuk D. Penney T. O'Hare A. J. Stokes J. Dolcos F. . (2012). Processing of emotional distraction is both automatic and modulated by attention: evidence from an event-related fMRI investigation. J. Cognit. Neurosci. 24, 1233–1252. 10.1162/jocn_a_00206 22332805 PMC4491634

[B118] Shafritz K. M. Collins S. H. Blumberg H. P. (2006). The interaction of emotional and cognitive neural systems in emotionally guided response inhibition. Neuroimage 31, 468–475. 10.1016/j.neuroimage.2005.11.053 16480897

[B119] Sitges C. González-Roldán A. M. Duschek S. Montoya P. (2018). Emotional influences on cognitive processing in fibromyalgia patients with different depression levels: an event-related potential study. Clin. J. Pain 34, 1106–1113. 10.1097/AJP.0000000000000637 29975206

[B120] Slaats-Willemse D. Swaab-Barneveld H. De Sonneville L. Van Der Meulen L. Buitelaar J. A. N. (2003). Deficient response inhibition as a cognitive endophenotype of ADHD. J. Am. Acad. Child Adolesc. Psychiatry 42, 1242–1248. 10.1097/00004583-200310000-00016 14560175

[B121] Song S. Zou Z. Song H. Wang Y. d'Oleire Uquillas F. Wang H. Chen H. (2016). Romantic love is associated with enhanced inhibitory control in an emotional stop-signal task. Front. Psychol. 7:215291. 10.3389/fpsyg.2016.01574 27826260 PMC5078777

[B122] Stockdale L. A. Morrison R. G. Silton R. L. (2020). The influence of stimulus valence on perceptual processing of facial expressions and subsequent response inhibition. Psychophysiology 57:e13467. 10.1111/psyp.13467 31454096

[B123] Storbeck J. Stewart J. L. Wylie J. (2024). Sadness and fear, but not happiness, motivate inhibitory behaviour: the influence of discrete emotions on the executive function of inhibition. Cognit. Emot. 38, 1160–1179. 10.1080/02699931.2024.2349281 38738654

[B124] Su H. Yang L. Cao H. Zhang J. Li Y. (2022). Effect of automatic emotional processing on response inhibition among heroin abstainers. PsyCh J. 11, 913–921. 10.1002/pchj.574 35701895

[B125] Sun L. Li J. Niu G. Zhang L. Chang H. (2020). Reactive aggression affects response inhibition to angry expressions in adolescents: an event-related potential study using the emotional go/no-go paradigm. Front. Psychol. 11:558461. 10.3389/fpsyg.2020.558461 33101129 PMC7556161

[B126] Todd R. M. Lee W. Evans J. W. Lewis M. D. Taylor M. J. (2012). Withholding response in the face of a smile: age-related differences in prefrontal sensitivity to Nogo cues following happy and angry faces. Dev. Cognit. Neurosci. 2, 340–350. 10.1016/j.dcn.2012.01.004 22669035 PMC6987687

[B127] van Holst R. J. van der Meer J. N. McLaren D. G. van den Brink W. Veltman D. J. Goudriaan A. E. . (2012a). Interactions between affective and cognitive processing systems in problematic gamblers: a functional connectivity study. PLoS One 7:e49923. 10.1371/journal.pone.0049923 23209619 PMC3509135

[B128] van Holst R. J. van Holstein M. van den Brink W. Veltman D. J. Goudriaan A. E. (2012b). Response inhibition during cue reactivity in problem gamblers: an fMRI study. PLoS One 7:e30909. 10.1371/journal.pone.0030909 22479305 PMC3316530

[B129] van Zutphen L. Siep N. Jacob G. A. Domes G. Sprenger A. Willenborg B. . (2020). Impulse control under emotion processing: an fMRI investigation in borderline personality disorder compared to non-patients and cluster-C personality disorder patients. Brain Imaging Behav. 14, 2107–2121. 10.1007/s11682-019-00161-0 31321661 PMC7647993

[B130] Verbruggen F. Aron A. R. Band G. P. Beste C. Bissett P. G. Brockett A. T. . (2019). A consensus guide to capturing the ability to inhibit actions and impulsive behaviors in the stop-signal task. eLife 8:e46323. 31033438 10.7554/eLife.46323 PMC6533084

[B131] Verbruggen F. De Houwer J. (2007). Do emotional stimuli interfere with response inhibition? Evidence from the stop signal paradigm. Cognit. Emot. 21, 391–403. 10.1080/0269993060062508134530318

[B132] Verbruggen F. Logan G. D. (2008). Response inhibition in the stop-signal paradigm. Trends Cognit. Sci. 12, 418–424. 10.1016/j.tics.2008.07.005 18799345 PMC2709177

[B133] Verbruggen F. Logan G. D. (2009). Proactive adjustments of response strategies in the stop-signal paradigm. J. Exp. Psychol. Hum. Percept. Perform. 35, 835–854. 10.1037/a0012726 19485695 PMC2690716

[B134] Verbruggen F. McLaren I. P. Chambers C. D. (2014). Banishing the control homunculi in studies of action control and behavior change. Perspect. Psychol. Sci. 9, 497–524. 10.1177/1745691614526414 25419227 PMC4232338

[B135] Vercammen A. Morris R. Green M. J. Lenroot R. Kulkarni J. Carr V. J. . (2012). Reduced neural activity of the prefrontal cognitive control circuitry during response inhibition to negative words in people with schizophrenia. J. Psychiatry Neurosci. 37, 379–388. 10.1503/jpn.110088 22617625 PMC3493093

[B136] Vercammen A. Skilleter A. J. Lenroot R. Catts S. V. Weickert C. S. Weickert T. W. . (2013). Testosterone is inversely related to brain activity during emotional inhibition in schizophrenia. PLoS One 8:e77496. 10.1371/journal.pone.0077496 24204845 PMC3814976

[B137] Verona E. Sprague J. Sadeh N. (2012). Inhibitory control and negative emotional processing in psychopathy and antisocial personality disorder. J. Abnorm. Psychol. 121, 498–510. 10.1037/a0025308 22288907

[B138] Wagner J. Wessel J. R. Ghahremani A. Aron A. R. (2018). Establishing a right frontal beta signature for stopping action in scalp EEG: implications for testing inhibitory control in other task contexts. J. Cognit. Neurosci. 30, 107–118. 10.1162/jocn_a_01183 28880766 PMC5908247

[B139] Wessel J. R. Aron A. R. (2015). It's not too late: the onset of the frontocentral P 3 indexes successful response inhibition in the stop-signal paradigm. Psychophysiology 52, 472–480. 10.1111/psyp.12374 25348645 PMC4830357

[B140] Wiemer J. Kurstak S. Sellmann F. Lindner K. (2023). Sexual stimuli cause behavioral disinhibition in both men and women, but even more so in men. Arch. Sex. Behav. 52, 1445–1460. 10.1007/s10508-022-02514-1 36694045 PMC10125947

[B141] Williams S. E. Lenze E. J. Waring J. D. (2020). Positive information facilitates response inhibition in older adults only when emotion is task-relevant. Cognit. Emot. 34, 1632–1645. 32677540 10.1080/02699931.2020.1793303 PMC7677201

[B142] Wilson K. M. De Joux N. R. Finkbeiner K. M. Russell P. N Helton W. S. (2016). The effect of task-relevant and irrelevant anxiety-provoking stimuli on response inhibition. Conscious. Cognit. 42, 358–365. 10.1080/02699931.2020.1793303 27149179

[B143] Windmann S. Chmielewski A. (2008). Emotion-induced modulation of recognition memory decisions in a Go/NoGo task: response bias or memory bias? Cognit. Emot. 22, 761–776. 10.1080/02699930701507899

[B144] Wolz I. Biehl S. Svaldi J. (2021). Emotional reactivity, suppression of emotions and response inhibition in emotional eaters: a multi-method pilot study. Appetite 161:105142. 10.1016/j.appet.2021.105142 33539909

[B145] Wöstmann N. M. Aichert D. S. Costa A. Rubia K. Möller H. J. Ettinger U. . (2013). Reliability and plasticity of response inhibition and interference control. Brain Cognit. 81, 82–94. 23174432 10.1016/j.bandc.2012.09.010

[B146] Xia X. Zhang G. Wang X. (2018). Anger weakens behavioral inhibition selectively in contact athletes. Front. Hum. Neurosci. 12:463. 10.1016/j.bandc.2012.09.010 30515088 PMC6255881

[B147] Xu M. Ding C. Li Z. Zhang J. Zeng Q. Diao L. . (2016a). The divergent effects of fear and disgust on unconscious inhibitory control. Cognit. Emot. 30, 731–744. 10.1080/02699931.2015.1027664 25861833

[B148] Xu M. Li Z. Ding C. Zhang J. Fan L. Diao L. . (2015). The divergent effects of fear and disgust on inhibitory control: an ERP study. PLoS One 10:e0128932. 10.1371/journal.pone.0128932 26030871 PMC4452620

[B149] Xu M. Li Z. Fan L. Sun L. Ding C. Li L. . (2016b). Dissociable effects of fear and disgust in proactive and reactive inhibition. Motivat. Emot. 40, 334–342. 10.1007/s11031-015-9531-9

[B150] Yang S. Luo W. Zhu X. Broster L. S. Chen T. Li J. . (2014). Emotional content modulates response inhibition and perceptual processing. Psychophysiology 51, 1139–1146. 10.1111/psyp.12255 24942597 PMC4383311

[B151] Yiend J. (2010). The effects of emotion on attention: a review of attentional processing of emotional information. Cognit. Emot. 24, 3–47. 10.1080/02699930903205698

[B152] You S. Lim C. E. Park M. Ryu S. Lee H. J. Choi J. M. . (2020). Response inhibition in emotional contexts in suicide ideators and attempters: evidence from an emotional stop-signal task and self-report measures. Psychol. Violence 10, 594–603. 10.1037/vio0000351

[B153] Yu F. Ye R. Sun S. Carretie L. Zhang L. Dong Y. . (2014). Dissociation of neural substrates of response inhibition to negative information between implicit and explicit facial Go/Nogo tasks: evidence from an electrophysiological study. PLoS One 9:e109839. 10.1371/journal.pone.0109839 25330212 PMC4199673

[B154] Yu F. Yuan J. Luo Y. J. (2009). Auditory-induced emotion modulates processes of response inhibition: an event-related potential study. Neuroreport 20, 25–30. 10.1097/WNR.0b013e32831ac9b1 18978645

[B155] Yu J. Hung D. L. Tseng P. Tzeng O. J. Muggleton N. G. Juan C. H. . (2012). Sex differences in how erotic and painful stimuli impair inhibitory control. Cognition 124, 251–255. 10.1016/j.cognition.2012.04.007 22658947

[B156] Yu J. Tseng P. Muggleton N. G. Juan C. H. (2015). Being watched by others eliminates the effect of emotional arousal on inhibitory control. Front. Psychol. 6:110988. 10.3389/fpsyg.2015.00004 25653635 PMC4299288

[B157] Yuan J. Tian Y. Huang X. Fan H. Wei X. (2019). Emotional bias varies with stimulus type, arousal and task setting: meta-analytic evidences. Neurosci. Biobehav. Rev. 107, 461–472. 10.1016/j.neubiorev.2019.09.035 31557549

[B158] Zhang J. Feng C. Mai X. (2016). Automatic emotion regulation in response inhibition: the temporal dynamics of emotion counter-regulation during a go/no-go task. Psychophysiology 53, 1909–1917. 10.1111/psyp.12754 27565763

[B159] Zhang J. Guan W. Chen X. Zhao Y. Liu P. (2023). Automatic emotion regulation prompts response inhibition to angry faces in sub-clinical depression: an ERP study. Biol. Psychol. 178:108515. 10.1016/j.biopsycho.2023.108515 36764597

[B160] Zhang L. Ye R. Yu F. Cao Z. Zhu C. Cai Z. . (2012). How does emotional context modulate response inhibition in alexithymia: electrophysiological evidence from an ERP study. PLoS One 7:e51110. 10.1371/journal.pone.0051110 23227242 PMC3515526

[B161] Zhang M. Wang S. Zhang J. Jiao C. Chen Y. Chen N. . (2020). The effects of subliminal goal priming on emotional response inhibition in cases of major depression. Front. Psychol. 11:542454. 10.3389/fpsyg.2020.542454 33414738 PMC7782471

[B162] Zhang W. Lu J. (2012). Time course of automatic emotion regulation during a facial Go/Nogo task. Biol. Psychol. 89, 444–449. 10.1016/j.biopsycho.2011.12.011 22200654

[B163] Zhang X. Jia H. Wang E. (2023). Negative inhibition is poor in sub-threshold depression individuals: evidence from ERP and a Go/No-go task. Psychiatry Res. Neuroimaging 331, 111638. 10.1016/j.pscychresns.2023.111638 37031674

[B164] Zhao D. Lin H. Xie S. Liu Z. (2019). Emotional arousal elicited by irrelevant stimuli affects event-related potentials (ERPs) during response inhibition. Physiol. Behav. 206, 134–142. 10.1016/j.physbeh.2019.04.005 30954488

[B165] Zheng Q. Yang T. X. Ye Z. (2020). Emotional stop cues facilitate inhibitory control in schizophrenia. J. Int. Neuropsychol. Soc. 26, 286–293. 10.1017/S1355617719001152 31694734

[B166] Zhuang Q. Xu L. Zhou F. Yao S. Zheng X. Zhou X. . (2021). Segregating domain-general from emotional context-specific inhibitory control systems-ventral striatum and orbitofrontal cortex serve as emotion-cognition integration hubs. NeuroImage 238:118269. 10.1016/j.neuroimage.2021.118269 34139360

